# Inactivation of *BRCA2* in human cancer cells identifies a subset of tumors with enhanced sensitivity towards death receptormediated apoptosis

**DOI:** 10.18632/oncotarget.7053

**Published:** 2016-01-28

**Authors:** Enrico N. De Toni, Andreas Ziesch, Antonia Rizzani, Helga-Paula Török, Sandra Hocke, Shuai Lü, Shao-Chun Wang, Tomas Hucl, Burkhard Göke, Christiane Bruns, Eike Gallmeier

**Affiliations:** ^1^ Department of Medicine II, Ludwig-Maximilians-University, 81377 Munich, Germany; ^2^ Department of Cancer Biology, University of Cincinnati, Cincinnati, Ohio 45267, USA; ^3^ Department of Gastroenterology and Hepatology, University of Prague, 14021 Prague 4, Czech Republic; ^4^ Department of Surgery, University of Magdeburg, 39120 Magdeburg, Germany; ^5^ Department of Gastroenterology, Philipps University Marburg, 35043 Marburg, Germany

**Keywords:** apoptosis, BRCA2, gene targeting, targeted therapy, TRAIL

## Abstract

**Purpose:**

DNA repair defects due to detrimental *BRCA2*-mutations confer increased susceptibility towards DNA interstrand-crosslinking (ICL) agents and define patient subpopulations for individualized genotype-based cancer therapy. However, due to the side effects of these drugs, there is a need to identify additional agents, which could be used alone or in combination with ICL-agents. Therefore, we investigated whether *BRCA2*-mutations might also increase the sensitivity towards TRAIL-receptors (TRAIL-R)-targeting compounds.

**Experimental design:**

Two independent model systems were applied: a *BRCA2* gene knockout and a *BRCA2* gene complementation model. The effects of TRAIL-R-targeting compounds and ICL-agents on cell viability, apoptosis and cell cycle distribution were compared in *BRCA2*-proficient versus-deficient cancer cells *in vitro*. In addition, the effects of the TRAIL-R2-targeting antibody LBY135 were assessed *in vivo* using a murine tumor xenograft model.

**Results:**

*BRCA2*-deficient cancer cells displayed an increased sensitivity towards TRAIL-R-targeting agents. These effects exceeded and were mechanistically distinguishable from the well-established effects of ICL-agents. *In vitro*, ICL-agents expectedly induced an early cell cycle arrest followed by delayed apoptosis, whereas TRAIL-R-targeting compounds caused early apoptosis without prior cell cycle arrest. *In vivo*, treatment with LBY135 significantly reduced the tumor growth of *BRCA2*-deficient cancer cells in a xenograft model.

**Conclusions:**

*BRCA2* mutations strongly increase the *in vitro*- and *in vivo*-sensitivity of cancer cells towards TRAIL-R-mediated apoptosis. This effect is mechanistically distinguishable from the well-established ICL-hypersensitivity of *BRCA2*-deficient cells. Our study thus defines a new genetic subpopulation of cancers susceptible towards TRAIL-R-targeting compounds, which could facilitate novel therapeutic approaches for patients with *BRCA2*-deficient tumors.

## INTRODUCTION

The DNA repair system represents a central mechanism of cellular homeostasis facilitating the detection and consequent repair of exogenous and endogenous DNA damage, thus preventing the perpetuation of potentially detrimental mutations and cancer formation. It consists of multiple DNA repair mechanisms which, depending on the recognized DNA damage pattern and cell cycle phase, activate different DNA repair pathways [1, 2]. Upon recognition of DNA damage, a complex system of events is triggered to restore genetic integrity. However, when DNA damage accumulates beyond the capability of the DNA repair system, apoptosis is triggered [3].

The Fanconi Anemia (FA) pathway represents the central DNA repair pathway for homologous recombination and is activated in response to stalled replication forks during the S-phase of the cell cycle [4]. Inactivation of this pathway occurs in patients suffering from the rare recessive disorder FA and is caused by bi-allelic germline mutations in one of at least 15 FA genes [5]. In addition, FA pathway-inactivation occurs at low frequency in various cancer entities among the general (non-FA) population, indicating a selective advantage during carcinogenesis, either through an early malignant transformation-promoting step due to an increased mutation rate or through an increased cellular tolerance towards abnormal DNA replication [6]. For example, proximal FA pathway inactivation due to rare mutations in *FANCC* or *FANCG* has been reported in pancreatic cancer [7, 8] and epigenetic inactivation of *FANCF* was found in a large variety of different tumors, including bladder cancer [9], breast cancer [10], cervical cancer [11], head and neck cancer [12], lung cancer [12] and ovarian cancer [13]. Even more prominently, inactivation of the distal FA pathway through mutations in the *BRCA2 (FANCD1)* gene has been reported in breast cancer [14] (familial cases [15–17]), pancreatic cancer [18, 19] and ovarian cancer [20], among others. Due to the well-established hypersensitivity of FA pathway-deficient tumor cells towards DNA-damaging ICL-agents, FA gene defects define patient subpopulations for individualized genotype-based therapies [21–23]. However, due to the side effects of these agents, there is a need to identify additional agents eliciting FA hypersensitivity, which could then be applied either alone or in combination with ICL-agents [23]. This concept was recently substantiated by reports of strong clinically responses of FA pathway-deficient cancers towards ICL-agents and PARP-inhibitors [24–28].

The two functional receptors for TRAIL, TRAIL-receptor-1 (TRAIL-R1) and TRAIL-receptor-2 (TRAIL-R2), are expressed in most human tissues and tumors and possess the particular ability to trigger apoptosis in cancer cells but not in non-malignant cells [29]. This tumor-selective pro-apoptotic effect of TRAIL-R stimulation is thought to reflect the physiological role played by the TRAIL-system during tumor-surveillance, which is regulated by the immune-mediated clearance of malignant and metastatic cells during the development of tumors. This function is supported by studies showing a correlation between loss of TRAIL-R-expression, poor prognosis and tumor recurrence [30–33] and by *in vivo* studies showing that TRAIL knockout (KO) mice exhibit enhanced primary tumor and metastasis formation [34]. Thus, TRAIL represents a promising novel anti-cancer therapy. Many types of recombinant TRAIL or agonistic antibodies targeting TRAIL-R have been made available for clinical use [29] and are currently tested in clinical trials. However, none of the previously conducted trials with TRAIL-R-targeting compounds reached their endpoint of improving patients’ outcomes (reviewed in [35]). One possible explanation for the failure of such agents to reproduce the effects achieved in preclinical experiments could be represented by the heterogeneity of the distribution of cell surface-bound cell receptors, as we previously suggested [31–33]. This idea seems to be supported by a very recent clinical trial showing that TRAIL-R2 imaging with radioactively labelled tigatuzumab (CS-1008) is predictive of clinical benefit in the treatment of patients affected by metastatic colorectal cancer [35]. In addition, the existence of intracellular mechanisms of resistance to TRAIL are likely limiting the clinical efficacy of these agents [36].

*BRCA2*-mutations are known to confer chemosensitivity to ICL-agents. Although this effect is known to be mediated by cell cycle jamming in consequence of impaired DNA repair, apoptosis is eventually triggered in these cells. In the present paper we assessed the possibility that *BRCA2*-deficient cells could thus display enhanced sensitivity towards the action of TRAIL in order to define a subpopulation of tumors highly susceptible to TRAIL-induced apoptosis which would be highly likely to respond to the clinical application of TRAIL-R targeting compounds. This hypothesis was tested first *in vitro* applying a *BRCA2* gene knockout (KO) model of the colorectal cancer cell line DLD1 [37] and a *BRCA2* gene complementation model of the *BRCA2*-mutant pancreatic cancer cell line CAPAN1 [38]. The *in vitro* results were consecutively validated *in vivo* using LBY in a murine xenograft model of *BRCA2*-deficient cancer cells. In addition, the underlying mechanisms of the observed hypersensitivity of *BRCA2*-deficient cancer cells towards apoptosis-inducing agents were explored.

## RESULTS

### Genetic inactivation of *BRCA2* enhances the susceptibility of cancer cells towards TRAIL-R-mediated apoptosis

To assess the effects of *BRCA2* status on the sensitivity towards TRAIL-R-mediated apoptosis, proliferation assays were performed upon administration of recombinant human TRAIL in parental *BRCA2* wild type DLD1 colon cancer cells (termed DLD1), heterozygote *BRCA2*^+/−^ KO cells (clone termed A9A2) and homozygous *BRCA2*^−/−^ KO cells (two independently established clones termed A10-1 and A10-3, respectively). As a control, the cells were treated with the ICL-agent MMC, for which the specific hypersensitivity of *BRCA2*-deficient cells is well-established.

**Figure 1:**
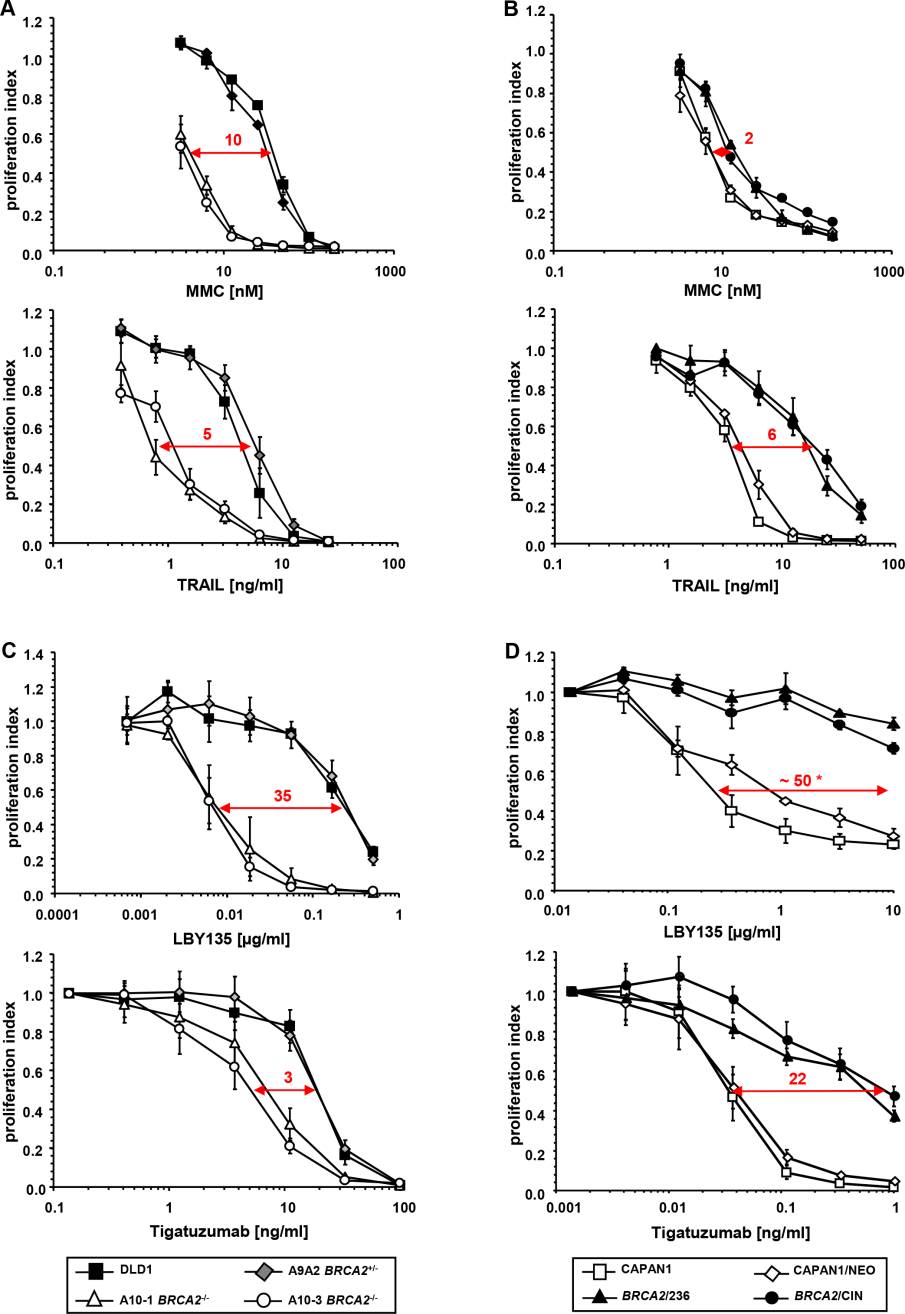
Genetic *BRCA2* inactivation enhances the sensitivity of cancer cells towards TRAIL-R-mediated apoptosis (**A**) Proliferation assays of *BRCA2*-proficient DLD1 and corresponding *BRCA2*^+/−^ A9A2 cells versus two corresponding homozygously *BRCA2*-deleted clones (A10-1 *BRCA2*^−/−^ and A10-3 *BRCA2*^−/−^) after treatment with the indicated agents. (**B**) Confirmatory proliferation assays of *BRCA2*-deficient parental (CAPAN1) and empty vector-transfected (CAPAN1/NEO) pancreatic cancer cells versus two *BRCA2*-complemented cell clones CAPAN1/CIN and CAPAN1/236 after treatment with the indicated agents. All experiments were performed in triplicate with error bars representing SEM from three independent experiments. * = extrapolated value. (**C, D**) Effects of *BRCA2*-status on cell viability upon administration of the clinically viable TRAIL-R2-targeting agonistic antibodies LBY-135 and tigatuzumab in *BRCA2*-proficient and *BRCA2*-deficient DLD1 cells (C) and in *BRCA2*-proficient and *BRCA2*-deficient CAPAN1 cells (D).

Upon treatment with MMC, we expectedly observed a strongly reduced proliferation rate in the two *BRCA2*^−/−^cell clones A10-1 and A10-3 as compared to the parental *BRCA2*^+/+^ and heterozygote *BRCA2*^+/−^ A9A2 cells (IC50 ratio approx. 10). A similar pattern was observed upon administration of TRAIL, which also caused a dose-dependent proliferation decrease in both *BRCA2*^−/−^ clones as compared to control cells (IC50 ratio approx. 5) (Figure 1A). Similar results were achieved after treatment with the FAS-agonistic antibody anti-Apo-1, which shows that the effects of *BRCA2*-status are not limited to TRAIL-R stimulation, but affect mechanisms of the activation of apoptosis common to other receptors of the extrinsic signaling pathway (Figure S1).

### Susceptibility towards TRAIL-R-mediated apoptosis is decreased by re-expression of BRCA2 in *BRCA2*-deficient cancer cells

To rule out cell line- or method-dependent phenomena, the data obtained in DLD1 cells were confirmed in a *BRCA2*-complementation model established by Wang et al. [38], employing the pancreatic cancer cell line CAPAN1. This cell line harbors the naturally occurring, inactivating *BRCA2* 6174delT frameshift mutation accompanied by loss of the second allele, the severe impact of which on *BRCA2* function has previously been extensively characterized [39]. In our experiments, parental CAPAN1 cells (termed CAPAN1) and empty-vector transfected cells (termed CAPAN1/NEO) were compared with two independently established CAPAN1 cell clones complemented by stably transfected *BRCA2* (termed BRCA2/236 and BRCA2/CIN). In these cells, re-expression of *BRCA2* decreased the sensitivity towards MMC as well as towards TRAIL (Figure 1B). However, the changes towards MMC were less obvious than those observed in the DLD1 model (IC50 ratio approx. 2), which might be attributable to the different experimental approaches (stable *BRCA2* overexpression in the *BRCA2*-deficient CAPAN1 line versus complete *BRCA2* KO in the *BRCA2*-proficient DLD1 line), a phenomenon previously observed in other studies [22, 23].

### *BRCA2* inactivation enhances the sensitivity of cancer cells towards TRAIL-R agonistic antibodies

To assess the potential clinical relevance of TRAIL-R targeting in *BRCA2*-deficient tumors, experiments were conducted in both the *BRCA2* KO DLD1 as well as the *BRCA2*-complementation CAPAN1 models using LBY135 [40] and tigatuzumab [41], two TRAIL-R2-targeting antibodies which have been recently made available for clinical application. These experiments clearly confirmed the enhanced susceptibility of *BRCA2*-deficient cells towards TRAIL-R-stimulation (Figure 1C and 1D). Of note, *BRCA2*^−/−^ cell clones displayed a 35-fold increased sensitivity towards LBY135, an effect significantly exceeding the well-established effects of all common DNA-ICL agents [21, 37]. This agent was thus expected to exert particularly strong effects in the experimental *in vivo* setting. Consequently, LBY135 was used for subsequent experiments.

### siRNA-mediated *BRCA2* knockdown enhances the sensitivity of cancer cells towards TRAIL-R-mediated apoptosis

**Figure 2:**
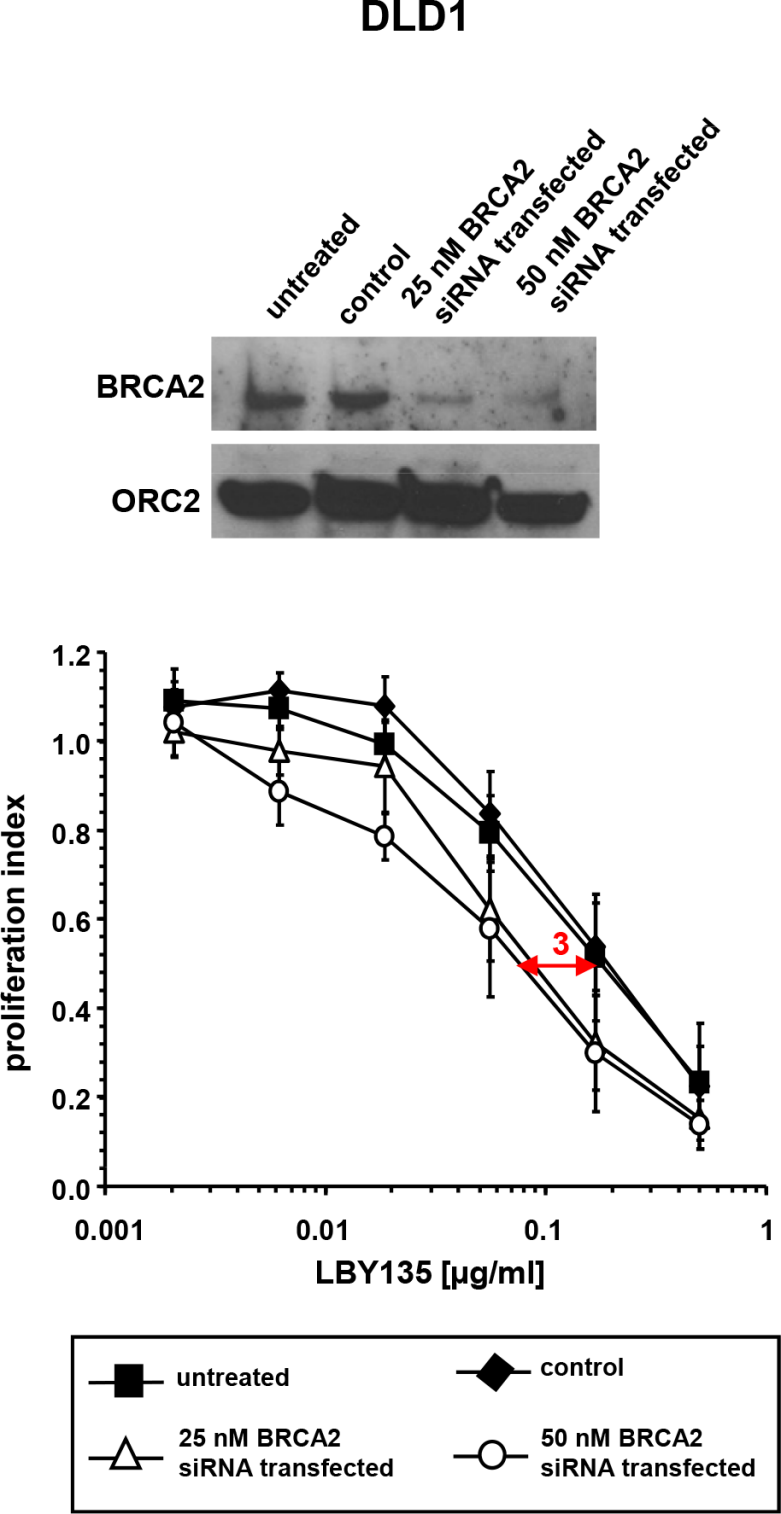
siRNA-mediated *BRCA2* knockdown enhances the sensitivity of cancer cells towards TRAIL-R-mediated apoptosis Western blot analysis of *BRCA2* expression levels upon siRNA-mediated knockdown of untreated, control- and *BRCA2*-siRNA-transfected DLD1 cells (upper panel). Corresponding proliferation assays after treatment with LBY135 (lower panel). Experiments were performed in triplicate with error bars representing SEM from three independent experiments.

To rule out clonal artefacts potentially occurring in gene KO and gene complementation models, RNA-interference experiments using *BRCA2*-siRNA were conducted in DLD1 cells. At 72 h after transfection with BRCA2-siRNA at 25 or 50 nM, respectively, LBY135 treatment resulted in a small but significant decrease of cell viability as compared to control-transfected cells (IC50 ratio approx. 3 fold) (Figure 2). The quantitatively smaller effects observed in the *BRCA2* siRNA model as compared to the *BRCA2* KO model are likely attributable to the incomplete protein depletion upon siRNA, an effect previously observed by us [22, 23].

### TRAIL-R stimulation causes early onset of apoptosis without preceding cell cycle arrest in *BRCA2*^−/−^ cells

**Figure 3:**
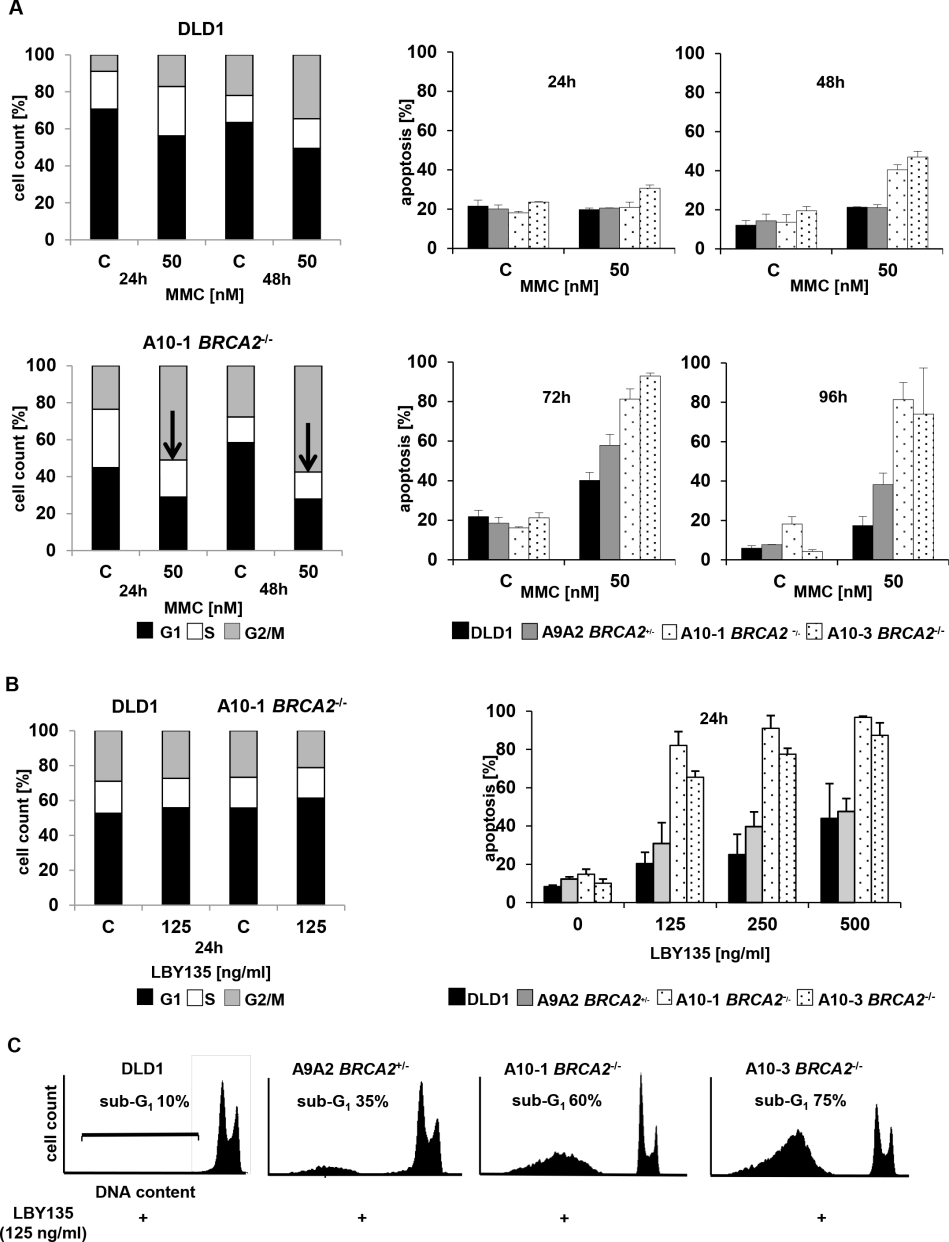
TRAIL-R stimulation causes early onset of apoptosis without concomitant cell cycle arrest in *BRCA2*^−/−^ cells (**A**) Cell cycle profiles (left panels) and subG1 fraction (right panels) of parental DLD1 cells (upper panels) versus *BRCA2*^−/−^ A10-1 cells (lower panels), treated with MMC at 50 nM or left untreated and assessed at the indicated time points. Arrows indicate samples displaying a significant G2/M arrest. (**B**) Cell cycle distribution (left panel) and subG1 fraction (right panel) of parental DLD1 cells versus *BRCA2*^−/−^ A10-1 cells, treated with LBY135 at the indicated concentrations or left untreated, assessed at 24 h after treatment. (**C**) Cell cycle profiles displaying representative results from one of at least three experiments derived from (B).

To analyze the mechanistic basis of the increased susceptibility of *BRCA2*-deficient cancer cells towards TRAIL-R-targeting agents, cell cycle arrest and apoptosis were determined upon treatment with LBY135 and the results compared to those observed upon treatment with MMC. First, cell cycle profiles were assessed by FACS analysis after administration of MMC or LBY135 in *BRCA2*-deficient vs. *BRCA2*-proficient cells. Then, apoptosis rates were assessed by measuring sub-G1 events, which correspond to the fraction of cells showing nuclear fragmentation in consequence of apoptotic death. Hypersensitivity of FA pathway-deficient cells towards ICL-agents is known to be associated with a late S-phase/G2/M arrest [7, 21]. Accordingly, MMC treatment of *BRCA2*^−/−^ cells expectedly led to a dose- and time-dependent early increase of the cell fraction in G2/M at 24 and 48 h as compared to control DLD1 cells (Figure 3A, left panels). G2/M-arrest was followed by apoptosis at 48 h, which progressively increased and peaked at 72 h after treatment (Figure 3A, right panel). In contrast, LBY135 caused apoptosis as early as 24 h after treatment without cell cycle arrest specifically in *BRCA2*^−/−^ cells (Figure 3B). Representative cell cycle profiles are shown (Figure 3C).

### DNA fragmentation and caspase cleavage confirms increased apoptosis in *BRCA2*^−/−^ cells

**Figure 4:**
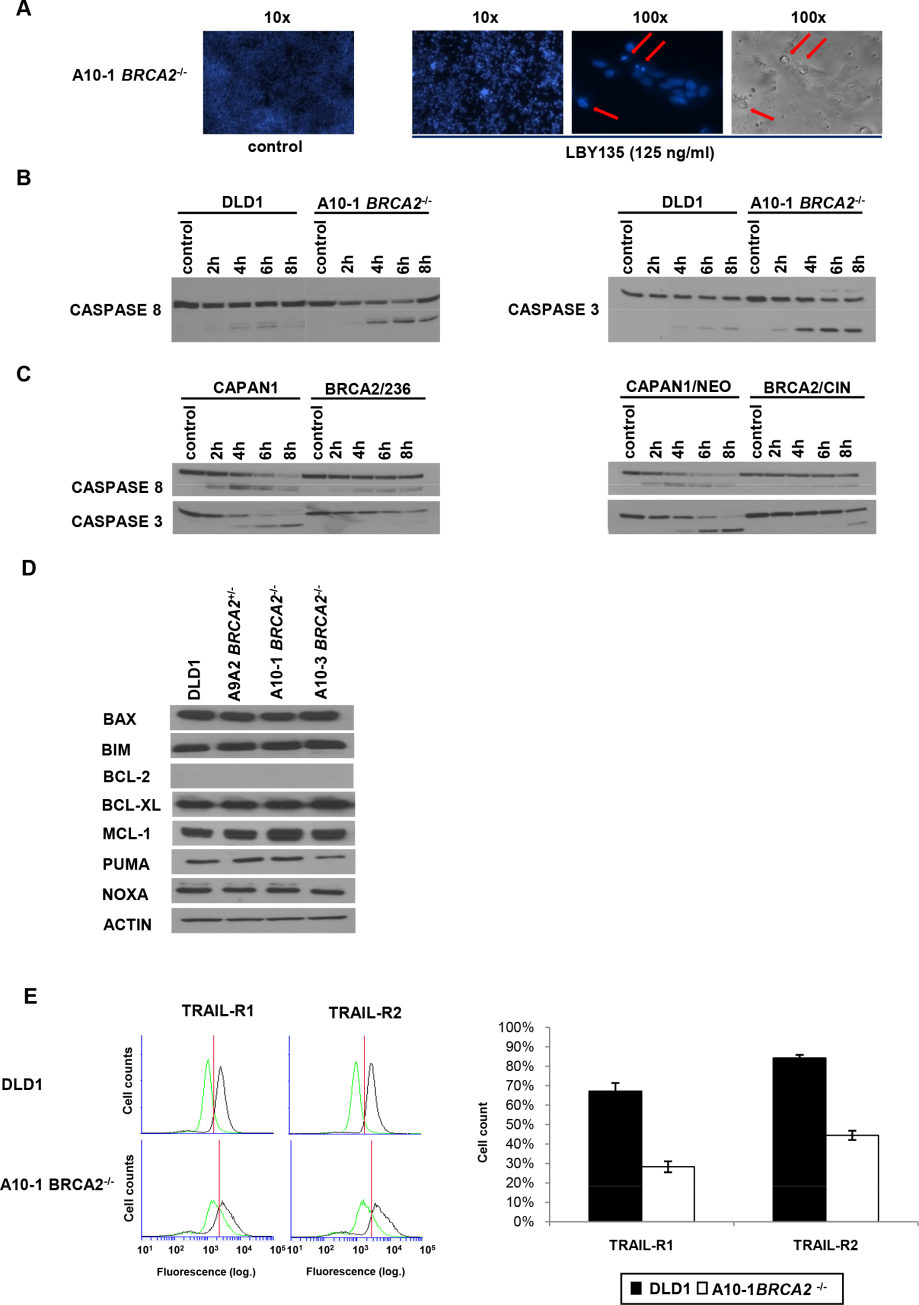
Apoptosis and CASPASE8 recruitment in *BRCA2*^−/−^ cells are not dependent on the mitochondrial pathway or on the regulation of TRAIL-receptors (**A**) Typical features of apoptosis (chromatin condensation and nuclear fragmentation) in DLD1 *BRCA2*^−/−^ A10-1 cells 24 h after treatment with LBY135 at 125 ng/ml, as assessed by fluorescence microscopy after Hoechst staining. (**B** + **C**) Western blotting to detect caspase 8 and caspase 3 cleavage upon treatment with LBY at 125 ng/ml at the indicated time points in parental DLD1 versus *BRCA2*^−/−^ A10-1 cells (B) and in *BRCA2*-deficient parental (CAPAN1) and empty vector-transfected (CAPAN1/NEO) cancer cells versus two *BRCA2*-complemented cell clones (CAPAN1/CIN and CAPAN1/236) (C). (**D**) Western blotting to assess the baseline expression levels of the indicated regulators of the mitochondrial pathway in *BRCA2*-proficient versus *BRCA2*-deficient DLD1 cells. BCL-2 was detectable in control cell lines (not shown) but not in DLD1 cells. (**E**) FACS analysis of surface receptor staining of TRAIL-R1 and TRAIL-R2 in *BRCA2*-proficient versus *BRCA2*-deficient DLD1 cells.

Apoptosis was validated by demonstration of massive DNA fragmentation as a typical apoptosis feature (Figure 4A) and increased cleavage of CASPASE3 (Figure 4B) in *BRCA2*^−/−^ cells after treatment with LBY135. Additionally, increased cleavage was also observed for CASPASE8 in *BRCA2*^−/−^ cells, suggesting that increased apoptosis occurs as consequence of the activation the death-inducing signaling complex (DISC) upon TRAIL-R-stimulation. This pattern of increased caspase cleavage was confirmed in the *BRCA2*-complementation CAPAN1 model (Figure 4C).

Since TRAIL-R-stimulation could result in necroptosis under certain circumstances [42], *BRCA2*^−/−^ and control DLD1 cells were additionally incubated with LBY135 with or without the addition of necrostatin to block necrosis induction. However, addition of necrostatin did not affect cell morphology or apoptosis rates (data not shown).

### TRAIL-R-induced apoptosis is not mediated by the mitochondrial apoptotic pathway in *BRCA2*-deficient cells

Since apoptosis triggered in response to DNA damage is typically triggered by alteration of the balance between pro- and anti-apoptotic factors that regulate mitochondrial polarisation [43], we next analyzed the baseline protein expression levels of principal mediators of the mitochondrial signaling pathway. However, except for a slight reduction of the expression of the pro-apoptotic molecule PUMA, which was observable only in the *BRCA2*^−/−^ A10-3 but not the *BRCA2*^−/−^ A10-1 clone (thus likely reflecting a clonal phenomenon), no significant changes in any of the tested regulators of the intrinsic apoptotic pathway were detected in *BRCA2*-proficient or -deficient cells, regarding both baseline expression (Figure 4D) and expression upon TRAIL-R stimulation (data not shown). Importantly, FACS analyses showed that membrane staining of TRAIL-R1 and -R2 did not differ in these cell lines (Figure 4E). These data suggest that the increased susceptibility of *BRCA2*-deficient cells towards TRAIL-R-targeting compounds specifically potentiates TRAIL-R-signaling and caspase 8 recruitment rather than enhancing downstream mitochondrial apoptotic signaling.

### *BRCA2*-deficiency delays tumor growth upon administration of LBY135 in a murine tumor xenograft model *in vivo*

**Figure 5:**
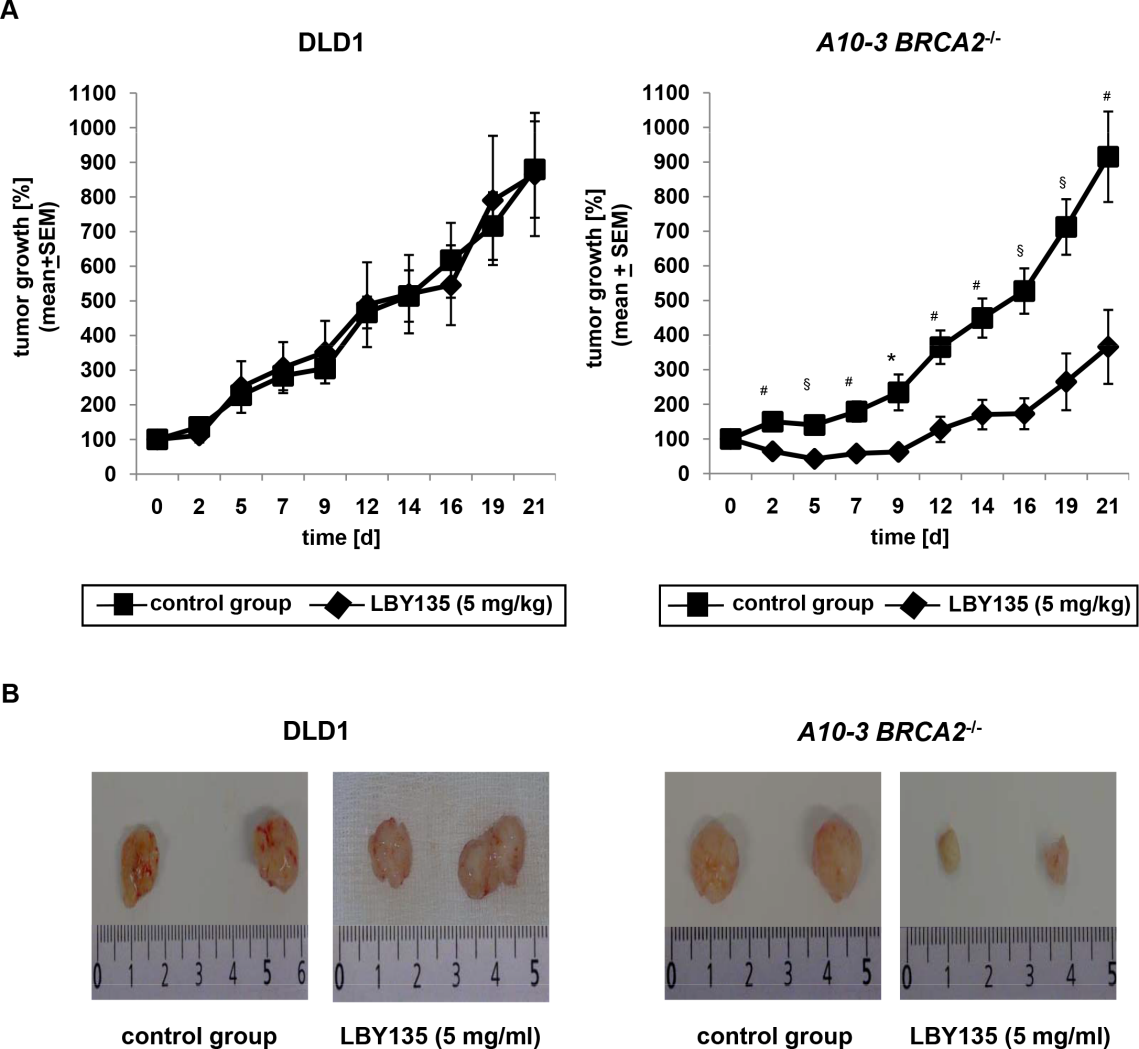
*BRCA2*-deficiency delays tumor growth upon administration of LBY135 in a murine tumor xenograft model *in vivo* (**A**) Time course (21d) of tumor growth as assessed by repeated measurements of xenograft tumors in mice subcutaneously injected with parental DLD1 cells or A10-3 *BRCA2*^−/−^ cell clones, respectively, which were consecutively treated intraperitoneally thrice a week with either LBY135 at 5 mg/kg or vehicle. Values are expressed as mean and standard error of the size of tumors at the indicated time points expressed as percentage to the baseline dimensions. *t*-test: *p < 0.01, #*p* < 0.005, §*p* < 0.001. (**B**) Representative pictures from excised tumors derived from parental and *BRCA2*^−/−^ A10-3 DLD1 cells.

To assess whether the increased sensitivity of *BRCA2*-deficient cells towards TRAIL-R-targeting compounds observed *in vitro* was transferable to the *in vivo* setting, we applied a murine tumor xenograft model. Tumors from parental DLD1 or corresponding *BRCA2*^−/−^ cells were induced in nude mice by subcutaneous injection. Animals exhibiting a solid palpable tumor mass at the injection site 5 days after tumor inoculation were randomized to receive intraperitoneally thrice a week LBY135 at 5 mg/kg or vehicle over a time period of 21 days (parental DLD1 cells: control group: *N* = 10, LBY135-treated group: *N* = 9. *BRCA2*^−/−^ A10-3 cell clones: control group: *N* = 9; LBY135 group: *N* = 12). Tumor growth rate was assessed by repeated tumor size measurements of the lesions, using the initial tumor lesion as denominator. LBY135 treatment caused a strongly decreased tumor growth rate as compared to vehicle-treated tumors specifically in tumors originating from *BRCA2*^−/−^ cells (LBY135-treated *BRCA2*^−/−^ cells on d21: 130 ± 22 mm^3^ vs. baseline 46 ± 7 mm^3^ [3.7 ± 1.1 fold increase]; control-treated *BRCA2*^−/−^ cells on d21: 308 ± 34 mm^3^ vs. baseline 36 ± 3.4 [9 ± 1.3 fold increase] – *p* < 0.001) (Figure 5A right panel), whereas no significant differences were observed in tumors from parental cells (LBY135-treated *BRCA2*^+/+^ cells on d21: 719 ± 146 mm^3^ vs. baseline 88 ± 9 mm^3^ [8.7 ± 1.8 fold increase]; control-treated *BRCA2*^+/+^ cells on d21: 663 ± 113 mm^3^ vs. baseline 75 ± 7 [8.8 ± 1.4 fold increase] – *p* = 0.70) (Figure 5A left panel).

## DISCUSSION

Aiming at expanding the therapeutic exploitation of *BRCA2*-deficiency for cancer therapy, we investigated here whether deleterious *BRCA2* mutations might confer increased susceptibility towards TRAIL-R-targeting agents. Such compounds, which are known to induce apoptosis selectively in cancer cells, have been recently developed and are currently undergoing clinical investigation in multiple tumor entities [29, 44].

In agreement with previous studies [37], treatment of *BRCA2*-deficient cells with MMC caused early cell cycle arrest at 24 h after treatment, which was subsequently followed by apoptosis reaching a peak at 72 h. In contrast, apoptosis upon TRAIL-R stimulation was induced without preceding or concomitant cell cycle arrest already at 24 h, as demonstrated by caspase cleavage and early appearance of cellular apoptotic features. These data show that the increased susceptibility of *BRCA2*-deficient cancer cells towards TRAIL-R-mediated apoptosis occurs independently of a prior cell cycle arrest and can thus mechanistically be distinguished from the well-established effects of ICL-agents [37]. Of note, *BRCA2*-deficient cancer cells displayed a significantly stronger hypersensitivity towards TRAIL-R stimulation using LBY135 than towards the classical ICL-agent MMC in our studies.

Using a murine xenograft model, we were able to confirm these *BRCA2* genotype-dependent effects of TRAIL-R stimulation *in vivo*: tumor growth in mice was significantly delayed upon intraperitoneal administration of LBY135 specifically in *BRCA2*^−/−^ cells, while no significant differences in tumor growth kinetics were observed in parental DLD1 cells. Our *in vitro*- and *in vivo*-data thus clearly demonstrate a hypersensitivity phenotype of *BRCA2*-deficient cells towards TRAIL-R-stimulation, resembling and exceeding the effects of ICL-agents, and could serve as a platform for clinical trials applying TRAIL-R-agonistic antibodies specifically in patients with *BRCA2*-mutant cancers.

To assess the distinct mechanisms of apoptosis induction by MMC or TRAIL-R stimulation, respectively (i.e. delayed apoptosis as a consequence of DNA-damage and cell cycle arrest vs. direct apoptosis) we examined the two main mechanisms of apoptosis induction, namely the extrinsic (or receptor-mediated) and the intrinsic (or mitochondrial) apoptotic pathway. The extrinsic pathway is triggered in cancer cells by the stimulation of apoptotic membrane receptors like FAS and TRAIL-R [3] which lead to CASPASE8 activation and, in turn CASPASE3 cleavage.

In contrast, the intrinsic pathway is initiated by cell-internal stimuli including DNA repair errors, which can occur either spontaneously or upon administration of DNA-damaging agents [43]. This leads to a shift in the pro-/anti-apoptotic balance towards pro-apoptotic molecules (e.g. BAX, PUMA or NOXA), which permeabilize the mitochondrial membrane, or to the repression of anti-apoptotic molecules (e.g. BCL-2 and BCL-XL) which, conversely, lead to mitochondrial stabilization [36]. Characteristically, accumulation of unrepaired DNA damage can cause a cell cycle arrest in the G2/M phase and subsequent apoptosis. This is exemplified in our experiments by the observed early G2/M arrest followed by apoptosis in *BRCA2*-deficient cells upon MMC treatment. Similarly, a previous study has shown an increase in the BAX to BCL-2 ratio and elevated cytochrome c release from the mitochondria in doxorubicin-treated *BRCA2*-knockout mice [45]. Due to this role of the mitochondrial pathway in determining apoptosis in *BRCA2*-deficient cells, we hypothesized that the increased sensitivity of these cells towards TRAIL-R targeting agents could be due to the increased recruitment of the mitochondrial signalling pathway upon CASPASE8 cleavage. However, neither baseline- nor TRAIL-R stimulation-induced expression levels of principal regulators of the intrinsic apoptotic pathway (including BAX, BIM, BID, BCL2, BCL-XL, MCL-1, NOXA, PUMA) differed between *BRCA2*-proficient and *BRCA2*-deficient cells. The prompt increase of CASPASE8 cleavage upon stimulation of TRAIL-R-mediated apoptosis specifically in *BRCA2*-deficient cells, which do not differ in the baseline expression of membrane-bound TRAIL-receptors from their *BRCA2*-proficient counterparts, along with the lack of typical changes in the mitochondrial apoptosis pathway suggests that this hypersensitivity phenotype is mediated at the level of the formation of the death-inducing signaling complex (DISC) and CASPASE8 recruitment. Interestingly, as opposed to our results in *BRCA2*-deficient cells, a previous study showed that MMC sensitizes cancer cells to the pro-apoptotic effects of TRAIL by downregulating the antiapoptotic proteins BCL2, MCL-1 and BCL-XL while upregulating several pro-apoptotic proteins including BAX and BIM. In addition, MMC induced an increase of cell surface trafficking of both TRAIL receptors in this study [46]. Taken together, these data indicate that *BRCA2* exerts antiapoptotic functions, which appear to be independent from its previously described role within the DNA repair system or mitochondrial pathway activation and suggest a role for *BRCA2* as a regulator of DISC.

Interestingly, preliminary data on isogenic RKO cancer cells harbouring defects in the proximal FA pathway revealed increased TRAIL-sensitivity only upon *FANCG* inactivation, while *FANCC* deficient cells remained unaffected (our own unpublished results). These distinct and gene-specific functions of individual proximal FA genes [47] have previously been observed by us in regard to ionizing radiation [21] and clearly warrant further exploration.

Our study has also certain limitations. Despite providing mechanistic insights, particularly by confining the influence of *BRCA2* on apoptosis to the level of CASPASE8 recruitment or cleavage, the present work does not clarify in-depth how exactly *BRCA2* mediates the sensitivity towards TRAIL-R stimulation. Specifically, the mechanisms that lead to increased CASPASE8 recruitment need to be further elucidated, including the involvement of the caspase-antagonist FLIP [48], the mechanisms controlling TRAIL-R clustering (e.g. O-glycosylation) [49] or the roles of certain kinases such as PKC, as recently suggested [50]. Furthermore, future proof-of-principle *ex-vivo* experiments using primary tumor samples could help to further strengthen the translational relevance of our findings as a precondition prior to their clinical application.

In conclusion, our data demonstrate that *BRCA2*-deficiency - modeled by two independent experimental techniques - confers increased sensitivity towards TRAIL-R-stimulation in cancer cell lines *in vitro* and *in vivo*. These effects are mechanistically characterized through early onset of apoptosis without concomitant cell cycle arrest and appear not be related to the well-known functions of *BRCA2* during DNA-repair. Importantly, while FA pathway- and particularly *BRCA2*-dependent hypersensitivities have previously been convincingly shown to be highly specific, reliably reproducible in different model systems and restricted mainly to ICL-agents [21, 37] and PARP inhibitors [51, 52], our data now expand the therapeutically exploitable spectrum to also include TRAIL R-targeting agents, providing a pre-clinical basis for clinical trials of newly developed TRAIL-R-agonistic antibodies in genetically defined subsets of patients with *BRCA2*-deficient tumors. On the one hand, patients developing resistance towards ICL-agents could benefit from a second-line treatment with TRAIL-R targeting compounds. On the other hand, the combination of TRAIL-R targeting compounds with ICL-agents is expected to either enhance the effects of ICL-agents or facilitate a dose-reduction, thus mitigating their side effects [23, 53]. Therefore, future pre-clinical studies will need to assess whether ICL-agents and TRAIL R-targeting drugs used in combination will cause synergistic, additive or antagonistic effects in *BRCA2*-deficient tumors [53]. Finally, recent studies in pancreatic and other cancer tissue samples indicate that the sub-cellular localization of TRAIL receptors influences the cellular sensitivity towards TRAIL [31–33]. As the recent clinical investigation of radio-labelled tigatuzumab demonstrates [35], these drug combinations might be thus particularly effective in those subsets of patients with *BRCA2*-deficient tumors that display an uptake for TRAIL-R-targeting antibodies.

## MATERIALS AND METHODS

### Cell lines and reagents

LBY135 was kindly provided by Novartis (Nuernberg, Germany), tigatuzumab (CS-1008) by Daiichi-Sankyo Pharma Development (Edison NJ, USA). Protein A was added at 0.01 times the concentration of LBY135 or tigatuzumab to facilitate antibody crosslinking. Recombinant human TRAIL/TNFSF10, was purchased from R & D Systems (Minneapolis, USA), Anti-Apo 1–3 from Alexis/ENZO Life Sciences (Lörrach, Germany). Parental DLD1 cells along with the corresponding heterozygote *BRCA2*^+/−^ (termed A9A2) and homozygote *BRCA2*^−/−^ (termed A10-1, A10-3) were kindly provided by Thomas Hucl and originally developed by him and Eike Gallmeier in Scott Kern's laboratory (Johns Hopkins University, Baltimore/MD, USA) [37]. CAPAN1 cells along with the corresponding empty vector-transfected CAPAN1/NEO and two *BRCA2*-complemented CAPAN1/236 and CAPAN1/CIN cell clones were kindly provided by Mien-Chie Hung in July 2010 (M.D. Anderson Cancer Center, Houston, Texas) [38]. *BRCA2* gene knockout was tested and confirmed in DLD1 cells on the genetic level by direct sequencing directly upon arrival, on the protein level by western blotting and on the functional level by assessment of sensitivity towards MMC and RAD51 focus formation. Likewise, *BRCA2* gene complementation of CAPAN1 cells was confirmed on the genetic level by direct sequencing, on the protein level by western blotting and on the functional level by assessment of RAD51 focus formation. Extensive screening efforts earlier conducted by us and others have previously illustrated the specific hypersensitivity of *BRCA2*, *FANCG*- and/or *FANCC*-deficient cells towards ICL-agents [21, 37] and PARP inhibitors [51, 52] and demonstrated that these gene defects did not have any effects towards most other therapeutic agents, including gemcitabine [7, 37, 54], which was therefore used among several other agents as negative control in our experiments (data not shown). All cell lines were grown in DMEM supplemented with 10% fetal calf serum, L-glutamine and penicillin/streptomycin (PAA, Cölbe, Germany).

### Cell proliferation assays

1,000–1,500 cells were plated in 96-well plates, allowed to adhere and then treated with the indicated agents at the indicated concentrations. After 6 days, cells underwent osmotic lysis in 100 μl H2O. After addition of 0.5% Picogreen (Molecular Probes, Invitrogen, Karlsruhe, Germany), fluorescence was measured (Cytofluor Series 4000, Applied Biosystems, Darmstadt, Germany) and the proliferation index calculated, defining the untreated samples as 1. Three independent experiments were performed per agent, with each data point reflecting triplicate wells. Error bars represent standard error of the mean (SEM) from three experiments.

### Immunoblotting

Proteins were loaded on 10% SDS PAGE gels and separated for 15 minutes at 80 V and for 60 minutes at 140 V and then transferred onto PVDF membranes. After blocking for 1 h in TBST (tris-buffered saline with Tween-20) containing 5% milk or 1% milk and 1% bovine serum albumin, membranes were incubated overnight with the following primary antibodies: CASPASE3 (#9662), CASPASE8 (#9508), CASPASE 9 (#9508) and BCL-XL (#2764), purchased from Cell Signaling/New England Biolabs GmbH (Frankfurt am Main, Germany), β-ACTIN (#A5441) from Sigma Aldrich (Munich, Germany), Bcl-2 (#610539) and BCL-XL (#610746) from BD Biosciences (Heidelberg, Germany), MCL-1 (#sc-12756), BAD (#sc-8044), BAK (#sc-7873), PUMA (#sc-374223), and NOXA (#sc-30209) from Santa Cruz Biotechnology (Heidelberg, Germany), BAX (#ab7977) from Abcam (Cambridge, UK), BIM (#559685) from BD Pharmingen (Heidelberg, Germany), XIAP (#IMG-5770) from Novus Biologicals LLC (Littleton, USA). Corresponding secondary antibodies were used at concentrations of 1:10.000. Detection was performed using the Super Signal West Pico Chemiluminescent Substrate (Thermo Scientific #34077 – Schwerte, Germany). BRCA2 immunoblotting was performed using anti-BRCA2 antibody (#OP95, Merck Millipore, Schwalbach, Germany) and ORC-2 (rabbit anti-ORC-2, #559266, purchased by BD Pharmingen) as a loading control [55].

### FACS analysis of surface receptors

DLD1 and A10-1 *BRCA2*^−/−^ cell clones were detached from the plates by 1% Trypsin-EDTA, washed and incubated for 30 min. with the following monoclonal FITC-coupled antibodies: TRAIL-R1 (ALX-804-297F-T100), TRAIL-R2 (ALX-804-298F-T100) from Enzo Life Science (Loerach, Germany) and control IgG1 (BD Bioscience, Heidelberg, Germany).

### Apoptosis and cell cycle assays

For cell cycle and apoptosis analyses, 8.0 × 10^4^−1.5 × 10^5^ cells were seeded in 12-well plates and, after overnight incubation, treated with the indicated agents. After propidium iodide staining, fluorescence-activated cell sorting (FACS – Accuri C6 flow cytometer, BD Biosciences San Jose, CA USA) was performed as previously described [9]. Apoptosis was quantified by the fraction of cells with sub-diploid DNA content (Sub-G1) and confirmed morphologically by Hoechst 33342 staining and fluorescence microscopy (Zeiss, Jena, Germany).

### siRNA-mediated BRCA2 knockdown

Parental DLD1 cells were allowed to reach 30%–50% confluence and then incubated with oligofectamine (Invitrogen) and siRNA directed against *BRCA2* (Hs *BRCA2* 7, Qiagen, Hilden, Germany) or with oligofectamine alone, which served as control. siRNA were used at final concentrations of 25 nM and 50 nM, respectively. 72 h after transfection, the cells were used for proliferation assays and immunoblotting.

### *In vivo*-studies

For xenograft tumor induction, 8 week old nu/nu nude mice were injected in both flanks with either 10^6^ isolated DLD1 parental cells or with the corresponding *BRCA2*^−/−^ KO cells (A10-3 clone). Each group consisted of 12 animals. After 5 days, animals displaying tumor engraftment, i.e. solid palpable lesions at either one or both of the injection sites, were randomized to receive a solution of LBY135 at 5 mg/kg or vehicle intraperitoneally thrice a week. Tumor growth was monitored daily over a time period of 21 days through repeated assessment of tumor volume, determined through measurement of the two major tumor diameters with a caliper. These data were expressed for each cell line as the ratio of the tumor size to the initial tumor size (measured at day 5 after injection of tumor cells). All animal experiments were performed according to the guidelines of the German law for animal life protection and approved by the local ethics committee.

## SUPPLEMENTARY MATERIALS FIGURES


