# Supplementary material for: Inactivation of BRCA2 in human cancer cells identifies a subset of tumors with enhanced sensitivity towards death receptormediated apoptosis
Source: Oncotarget. 2016 Jan 28;7(8):9477–90. doi: 10.18632/oncotarget.7053 (PMC4891053; doi:10.18632/oncotarget.7053)
Supplement: Supplementary file 1 [file oncotarget-07-9477-s001.pdf]

Inactivation of *BRCA2* in human cancer cells identifies a subset of tumors with enhanced sensitivity towards death receptor-mediated apoptosis

Supplementary Materials

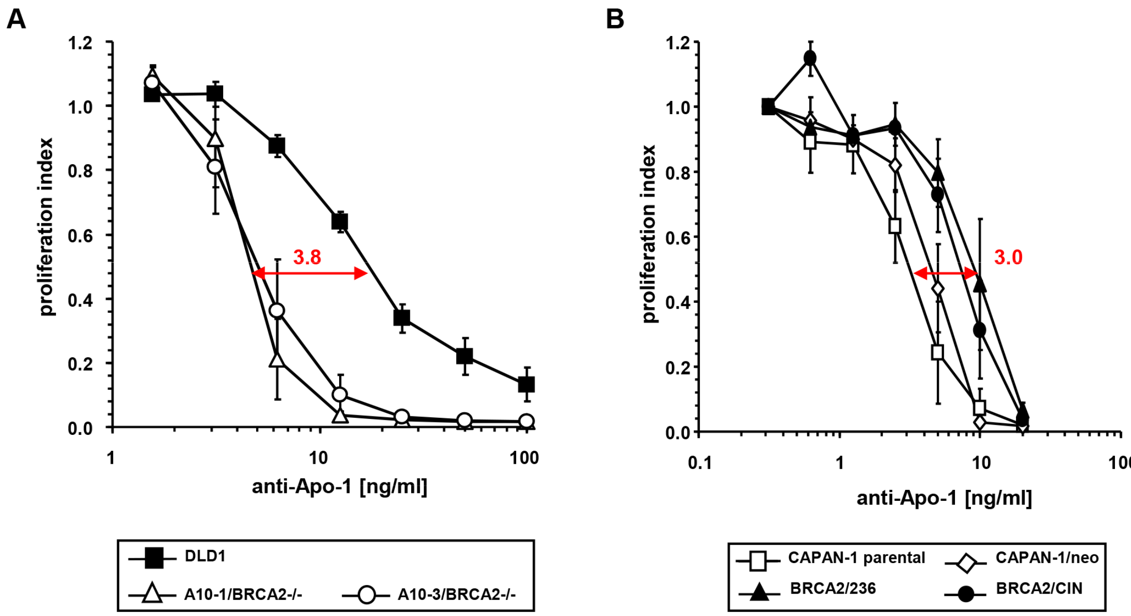

Supplementary Figure S1: Genetic *BRCA2* inactivation enhances the sensitivity of cancer cells towards FAS-mediated apoptosis. (**A**) Proliferation assays of parental *BRCA2*-proficient DLD1 versus two corresponding homozygously BRCA2-deleted clones (A10-1 $BRCA2^{-/-}$ and A10-3 $BRCA2^{-/-}$) after treatment with the FAS-agonistic antibody Anti-Apo-1. (**B**) proliferation assays of *BRCA2*-deficient parental (CAPAN1) and empty vector-transfected (CAPAN1/NEO) pancreatic cancer cells versus two *BRCA2*-complemented cell clones CAPAN1/CIN and CAPAN1/236 after treatment with anti-Apo-1.
